# Preoperative CT-Based Radiomics Combined With Nodule Type to Predict the Micropapillary Pattern in Lung Adenocarcinoma of Size 2 cm or Less: A Multicenter Study

**DOI:** 10.3389/fonc.2021.788424

**Published:** 2021-12-02

**Authors:** Meirong Li, Yachao Ruan, Zhan Feng, Fangyu Sun, Minhong Wang, Liang Zhang

**Affiliations:** ^1^ Department of Radiology, The First Affiliated Hospital, Zhejiang University, School of Medicine, Hangzhou, China; ^2^ Department of Radiology, Xiaoshan Hospital of Traditional Chinese Medicine, Hangzhou, China; ^3^ Department of Radiology, First Affiliated Hospital of Wannan Medical College, Wuhu, China; ^4^ Department of Radiology, Cancer Hospital of the University of Chinese Academy of Sciences (Zhejiang Cancer Hospital), Hangzhou, China

**Keywords:** lung adenocarcinoma, radiomics model, micropapillary pattern (MPP), multicenter, computed tomography

## Abstract

**Purpose:**

To construct an optimal radiomics model for preoperative prediction micropapillary pattern (MPP) in adenocarcinoma (ADC) of size ≤ 2 cm, nodule type was used for stratification to construct two radiomics models based on high-resolution computed tomography (HRCT) images.

**Materials and Methods:**

We retrospectively analyzed patients with pathologically confirmed ADC of size ≤ 2 cm who presented to three hospitals. Patients presenting to the hospital with the greater number of patients were included in the training set (n = 2386) and those presenting to the other two hospitals were included in the external validation set (n = 119). HRCT images were used for delineation of region of interest of tumor and extraction of radiomics features; dimensionality reduction was performed for the features. Nodule type was used to stratify the data and the random forest method was used to construct two models for preoperative prediction MPP in ADC of size ≤ 2 cm. Model 1 included all nodule types and model 2 included only solid nodules. The receiver operating characteristic curve was used to assess the prediction performance of the two models and independent validation was used to assess its generalizability.

**Results:**

Both models predicted ADC with MPP preoperatively. The area under the curve (AUC) of prediction performance of models 1 and 2 were 0.91 and 0.78, respectively. The prediction performance of model 2 was lower than that of model 1. The AUCs in the external validation set were 0.81 and 0.72, respectively. The DeLong test showed statistically significant differences between the training and validation sets in model 1 (p = 0.0296) with weak generalizability. There was no statistically significant difference between the training and validation sets in model 2 (p = 0.2865) with some generalizability.

**Conclusion:**

Nodule type is an important factor that affects the performance of radiomics predictor model for MPP with ADC of size ≤ 2 cm. The radiomics prediction model constructed based on solid nodules alone, can be used to evaluate MPP and may contribute to proper surgical planning in patients with ADC of size ≤ 2 cm.

## Introduction

With widespread use of high-resolution CT (HRCT), lung cancer is increasingly being detected at an early stage and small peripheral lung cancers are increasingly treated with surgical resection. Adenocarcinoma (ADC) is the most common lung cancer type. According to the World Health Organization classification of lung ADC (1), invasive adenocarcinoma (IAC) is divided into five pathological types, namely lepidic, acinar, papillary, micropapillary, and solid subtypes. The micropapillary pattern (MPP) is a marker for poor outcome (2–6). Lee (7) found that the overall survival and disease-free survival were worse in cases with minimal areas of MPP (accounting for 1-5% of the entire tumor). Since this adverse prognostic effect, identification of MPP may potentially be instructive for surgical plan and further aggressive adjuvant treatment.

Tumor size and MPP are important prognostic factors for surgical outcome in patients with early stage lung ADC. The use of limited resection has gradually increased for patients with non-small cell lung cancer with size ≤ 2 cm. Notably, MPP accounting for more than 5% of the entire tumor is an independent risk factor for recurrence and poor outcome of lung ADC with size ≤ 2 cm (3, 8, 9), suggesting that limited resection may not be the optimal surgical approach for such patients. Thus, preoperative confirmation of MPP (constituting> 5%) in ADC with size ≤ 2 cm has importance for selection of surgical procedure. Due to technical reasons, preoperative histological examination cannot be performed for many peripheral small tumors.

Radiomics can objectively and quantitatively analyze imaging features that reflect tumor heterogeneity. Previous studies showed that radiomics can be used to preoperatively detect ADC with MPP or MPP/solid pattern (10–18). However, previous reports of predicting MPP subtypes using radiomic analyses are with some limitations. First, previous studies have been conducted on the predictor MPP for T1-stage ADC (17, 18), including patients of tumors with size ≥ 2 cm. Because the preferred surgical procedures for ADC with sizes ≤ 2 cm or > 2 cm are different, prediction of MPP in ADC with size ≤ 2 cm can aid in determining the optimal resection method. In addition, previous studies considered only tumor size and overlooked different imaging characteristics between ground glass opacity (GGO) and solid nodule. These radiomics studies combined solid and GGO nodules but the unequal number of these two nodule types may have introduced bias in these study results. Furthermore, these studies were performed on small populations and lacked external validation.

Therefore, the purpose of our retrospective study was to develop an optimal radiomics model for preoperative prediction of MPP with ADC of size ≤ 2 cm. First, we included a large number of ADC patients with peripheral tumor size ≤ 2 cm from three institutions. Second, nodule type was used for stratification and it was combined with HRCT radiomics characteristics to construct two models. Model 1 included all nodule types (solid and GGO). Because most ADC with MPP of size ≤ 2 cm were solid (19), the GGO type was excluded for model 2; only solid nodules were used to construct model. Third, independent external validation was used to validate the two model’s generalization ability.

## Materials And Methods

### Patients

This was a retrospective study, which was approved by the institutional review board of the First Affiliated Hospital of Zhejiang University School of Medicine, the First Affiliated Hospital of Wannan Medical College and Zhejiang Cancer Hospital. Informed consent by the patients was waived by the hospital ethics committee. Patients diagnosed with ADC between September 2019 and January 2021 were selected from three study centers.

Patients were included in the study if they had a tumor with size on CT images < 2 cm, peripheral nodules on chest CT, no marked cavitation of lesions, histologic subtype without solid component, and surgery within 1 week of CT examination.

We excluded patients with history of neoadjuvant chemotherapy or radiotherapy, lung cancer surgery in the past 2 years, simultaneous multiple cancers, and patients with multifocal lesions.

We included 2,386 patients (mean age: 51.62 ± 13.13 years; range: 27–76 years) from Hospital 1 in this study. The proportion of female patients was 76.3%, 194 patients had MPP and 2,192 did not have MPP. Of the 2386 patients, 400 had solid nodule and 1,792 had GGO.

The independent external validation database consisted of 119 patients from the other two hospitals. The proportion of female patients was 66.4% and the average age was 60.56 ± 9.51 years (range: 29–80 years). There were 65 patients with MPP and 54 without MPP. Of the 119 patients, 80 had solid nodule and 39 had GGO.

**Figure 1:**
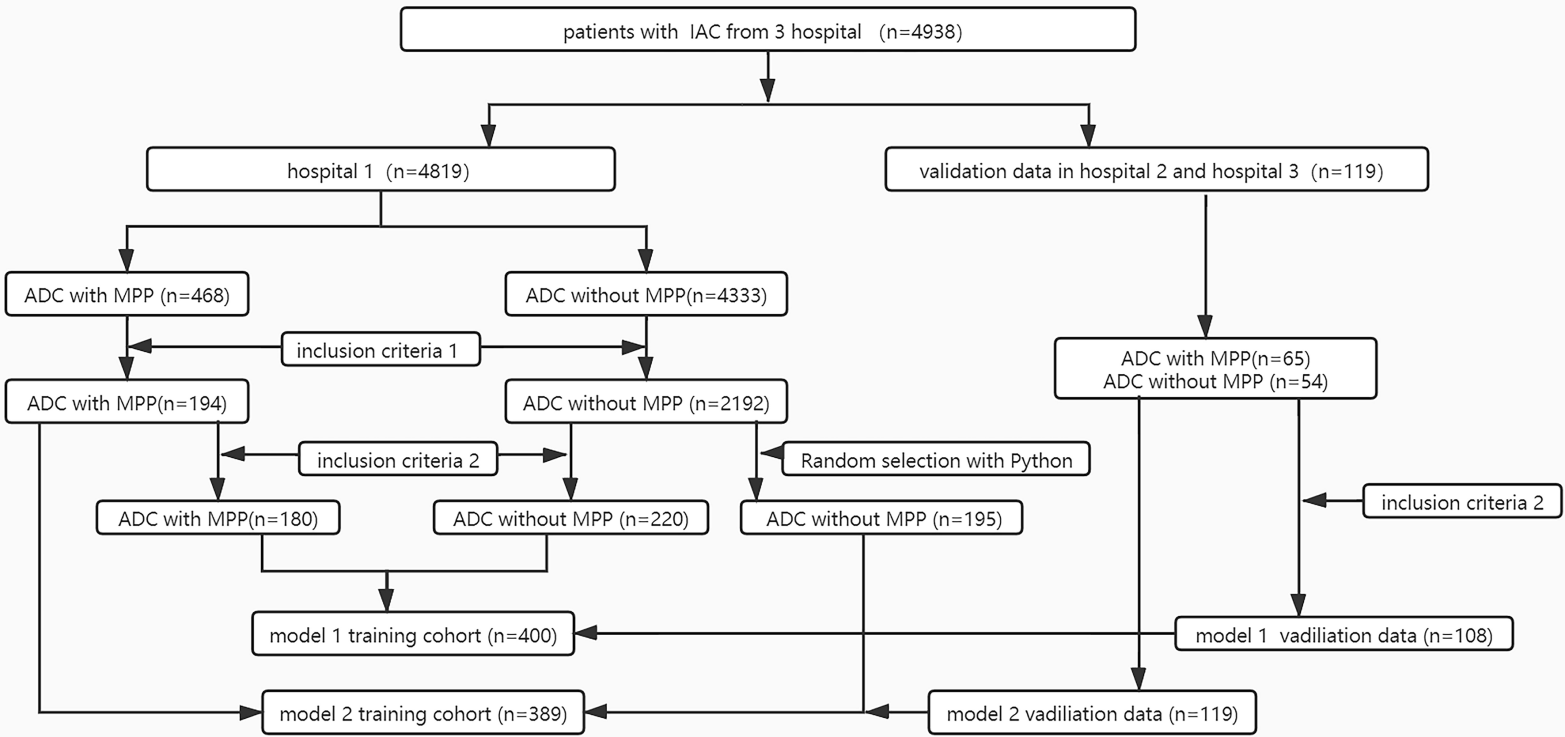
Flowchart selection patients of the two-model dataset. Inclusion criteria 1: tumor size measured in CT images <2 cm;2) the presence of peripheral nodules on chest CT; 3) the lesions without marked cavity;4) histologic subtype without solid pattern; Inclusion criteria 2: the nodule manifest as solid on chest CT.

To imbalance data numbers between the two groups, when model 1 (including GGO and solid nodules) was constructed, 2,192 ADC patients without MPP from hospital 1 were matched to 195 controls. Finally, Model 1 included 389 patients from hospital 1 and 119 patients from the other two hospitals in the training and validation sets, respectively. When constructing model 2 (only including solid nodules), 400 and 108 patients from hospitals 1 and the other two hospitals respectively, were included in the training and validation sets. **Figure 1** shows the model construction flow chart. The clinical variables were retrospectively reviewed from the electronic medical records.

### CT Imaging Acquisition

Pulmonary CT imaging was performed for all patients, using six CT scanners from Philips Healthcare (iCT 256 and 16-slice CT), Siemens Healthineers (64-slice CT), and GE (64-slice CT). All scans were acquired with a deep inhaled breath held in the supine position, without contrast. The scan covered from the thoracic inlet to bilateral adrenal glands. A sharp reconstruction algorithm was used. CT imaging parameters used in the three institutions were as follows: tube voltage of 100 or 120 kev, automatic tube current modulation; reconstructed slice thickness 1-3 mm. and reconstruction interval 0.625-1 mm. The images were displayed in two gray scales for interpretation of lung (width 1500 HU, level −430 HU) and mediastinal (width 200 HU, level 40 HU) windows.

### Histologic Evaluation and CT Features

Histological subtype was independently evaluated by two experienced radiologists and discrepancies were resolved through consensus. According to the 2015 World Health Organization classification of lung tumors (1), ADC histologic subtypes were recorded using a semiquantitative assessment of each subtype in 5% increments. In our study, according to the amount of micropapillary component, patients were divided into group 1 (ADC with MPP constituting > 5% of the entire tumor) and group 2 (ADC without MPP or MPP < 5% of the entire tumor). Based on the guidelines from the Fleischer society (20) nodule type (pure ground glass opacity, mixture ground glass opacity or solid) was determined by two radiologists on the lung window setting. In our study, the pure ground glass opacity (pGGO) and mixture ground glass opacity(mGGO) nodules were classified as GGO.

### Tumor Segmentation and Radiomics Feature Extraction

**Figure 2:**
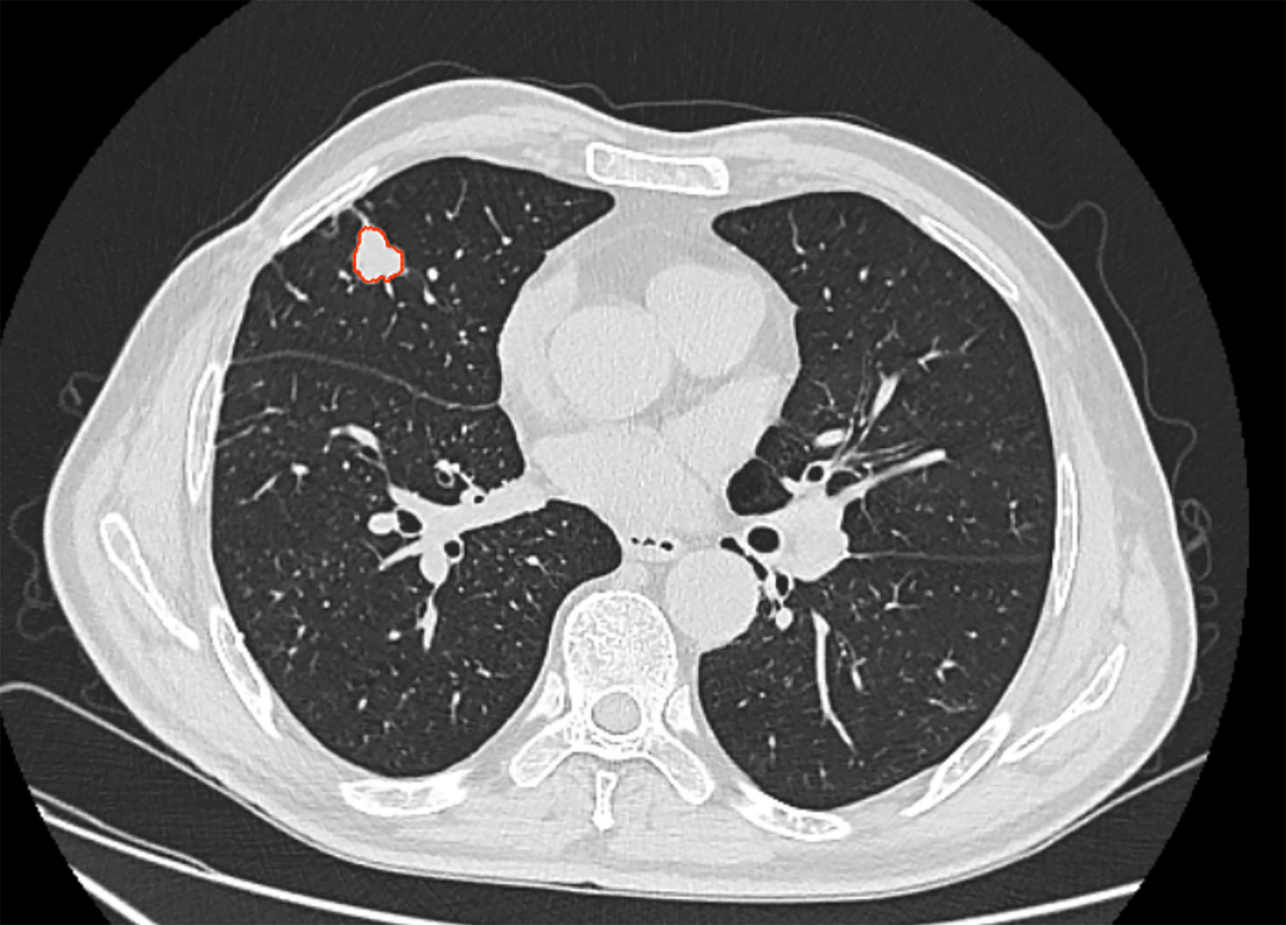
52 years-old patient, male, the lesion located in right middle lung. The area inside the red line represents the ROI for the tumor.

In this study, the Dr. Wise^®^ research platform was used for radiomics analysis. All patient images were downloaded and processed in the raw DICOM format and images were transferred to the post-processing workstation. One radiologist manually labeled the lesion region in thin-layer HRCT using the raw dataset (**Figure 2**) to avoid bronchovascular bundles and normal lung parenchyma. Then, the second radiologist confirmed the final regions of interest with consensus.

### Image Preprocessing

The radiomics features included first-order features, tumor morphologic features, gray-level co-occurrence matrix describing the tumor internal and surface textures, gray-level run-length matrix, gray-level size zone matrix, and gray level dependence matrix texture features. A total of 105 radiomics features were extracted from every ROI and Z-score standardization was performed.

### Feature Dimensionality Reduction and Model Construction

Spearman’s correlation analysis was performed for radiomics features using a correlation coefficient threshold of 0.8. Then, a tree-based algorithm was used for feature dimensionality reduction. The random forest method was used to construct two prediction models, based on the nodule types combined with radiomics features extracted from plain HRCT images. Model 1 contained solid nodules and GGO, while model 2 contained only solid nodules. Ten cross-validations were performed on the training set to analyze model stability. The ROC curve was used to evaluate the diagnostic performance of two models in predicting ADC with MPP in the training and validation sets. Finally, the generalizability of the two models was evaluated using the external validation set.

### Statistical Analysis

R software (version: 3.4.1; R Foundation for Statistical Computing, Vienna, Austria; http://www.Rproject.org) was used for the statistical analyses. Quantitative data were expressed as mean ± standard deviation (SD), while qualitative data were expressed as frequency (%). Qualitative variables (gender, nodule type, and ADC with/without MPP) were compared using the Chi-square test. Continuous variables (age) were evaluated using a two-sample t-test or Mann-Whitney U test.

The area under the ROC curve (AUC), 95% confidence interval (CI), accuracy, sensitivity, specificity, positive predictive value (PPV), and negative predictive value (NPV) of the two models were calculated for the training and validation sets. DeLong test was used to compare AUC differences between the training and validation sets for the two models. A two-tailed difference of p < 0.05 was deemed to be statistically significant.

## Result

### Dataset Characteristics

The clinical characteristics of the two model databases are summarized in **Tables 1**, **2**.

**Table 1:** Demographic and clinical characteristics of patients on different datasets of model 1.

Model 1	Training		P	validation		P
	MPP (n = 194)	Without MIP (n = 195)		MPP (n = 65)	Without MPP (n = 54)	
Age	61.8 ± 10.9	60.8 ± 11.1	0.25	60.5 ± 9.3	60.6 ± 9.9	0.29
Gender						0.045
Man	104 (54%)	56 (29%)	<0.001	31 (48%)	15 (32%)	
Woman	90 (46%)	139 (71%)		34 (51%)	39 (72%)	
Nodule type			<0.001			<0.001
Solid	180 (93%)	61 (31%)		60 (92%)	20 (37%)	
GGO	14 (7%)	134 (69%)		5 (8%)	34 (63%)	

**Table 2:** Demographic and clinical characteristics of patients on different datasets of model 2.

Model 2	Training		P	validation		P
	MPP (n=180)	Without MPP (n = 220)		MPP (n = 60)	Without MPP (n = 48)	
Age	62 ± 10.8	61.4 ± 10.9	0.28	61.1 ± 9.2	60.8 ± 9.2	0.19
Gender			<0.001			0.012
Man	96 (53%)	70 (32%)		31 (52%)	11 (29%)	
Woman	84 (47%)	150 (68%)		29 (48%)	37 (71%)	

Model 1 included 389 patients from the training set. ADC patients with MPP were aged 31–87 years (61.8 ± 10.9), 46% were females, 93% had solid nodules, and 7% had GGOs. ADC patients without MPP were aged 20–81 years (60.8 ± 11.1), 71% were females, 31% had solid nodules, and 69% had GGOs. There were 119 patients in the validation set. In the validation set, the age of ADC patients with MPP was 35–81 years (60.5 ± 9.3), 51% were females, 92% had solid nodules, and 8% had GGOs. ADC patients without MPP were aged 29–84 years (60.56 ± 9.91), 72% were females, 37% had solid nodules, and 63% had GGOs.

There were 400 patients in Model 2 from the training set. In the training set, ADC patients with MPP were aged 31–83 years (62 ± 10.8) and 47% were females. ADC patients without MPP were aged 26–83 years (61.4 ± 10.9) and 68% were females. There were 108 patients in the validation set. In the validation set, ADC patients with MPP were aged 35–81 years (61 ± 9.2) and 48% were females. ADC patients without MPP were aged 29–84 years (60.8 ± 9.2) and 68% were females.

No statistically significant difference was observed in the age between the training and validation sets in the two models, but statistically significant differences in gender were observed. The difference in solid nodule ratio was statistically significant between the two groups.

### Feature Selection

**Tables 3**, **4** shows the radiomics characteristics used in the two models. Eight radiomics characteristics were included in model 1: gray level matrix (GLDM; n = 4) and first order (n = 4). Eighteen optimal radiomics characteristics were included in model 2: gray level co-occurrence matrix (GLCM; n = 6); first order (n = 4), shape feature (n = 4), neighborhood gray tone difference matrix (NGTDM; n = 2) and gray level size zone matrix (GLSZM; n = 2).

**Table 3:** Selected radiomic features for the prediction model 1.

Feature class	Feature name	Feature coefficient	Weight
First order	Mean	0.207	1
GLDM	LDHGLE	0.1884	0.9104
First order	Energy	0.1824	0.8815
First order	10 Percentile	0.1016	0.491
First order	Minimum	0.0833	0.4027
GLDM	SDLGLE	0.0809	0.391
GLDM	LDLGLE	0.0784	0.3786
GLDM	Contrast	0.0779	0.3765

**Table 4:** Selected radiomic features for the prediction model 2.

Feature class	Feature name	Feature coefficient	Weight
First order	Energy	0.0911	1
GLCM	Imc2	0.0844	0.9271
GLCM	Imc1	0.0756	0.8296
GLCM	Dependence Non-Uniformity Normalized	0.0718	0.7886
shape	Sphericity	0.0657	0.7211
First order	Kurtosis	0.0583	0.6406
shape	Least Axis Length	0.0578	0.6347
GLCM	Correlation	0.0577	0.6335
GLCM	Joint Entropy	0.0519	0.5704
GLSZM	Large Area High Gray Level Emphasis	0.0507	0.5571
GLCM	Maximum Probability	0.0495	0.5433
First order	10 Percentile	0.0472	0.5184
GLSZM	SZN	0.047	0.5161
NGTDM	Busyness	0.0442	0.4849
First order	Minimum	0.0429	0.471
NGTDM	Coarseness	0.0406	0.4462
shape	Major Axis Length	0.032	0.3517
shape	Maximum 3D Diameter	0.0315	0.3463

GLCM, Gray Level Co-occurrence Matrix; GLDM, L Gray Level Dependence Matrix; GLSZM, Gray Level Size Zone Matrix; NGTDM, Neighborhood Gray Tone Difference Matrix; DHGLE, Large Dependence High Gray Level Emphasis; SDLGLE, Small Dependence Low Gray Level Emphasis; LDLGLE, Large Dependence Low Gray Level Emphasis; Dependence Non-Uniformity Normalized; SZN, Size Zone Non-Uniformity Normalized.

### Evaluation of Model Prediction Performance

The AUC values for the two radiomics models in training and validation cohort were shown in **Table 5**. The ROC curve results showed that model 1 had excellent preoperative prediction for ADC with MPP. In the training set, AUC was 0.91 (95% CI 0.88–0.94), accuracy was 0.79, sensitivity was 0.73, specificity was 0.86, PPV was 0.79, and NPV was 0.82. In the validation set, AUC was 0.82 (95% CI 0.74–0.89), accuracy was 0.83, sensitivity was 0.76, specificity was 0.88, PPV was 0.83, and NPV was 0.83.

**Table 5:** Predictive probabilities for the two radiomic model on the training and validation cohort.

		AUC_CI	Accuracy	Sensitivity	Specificity	PPV	NPV
Model 1: the lesion including solid, part-solid and GGO nodule							
	Training	0.91[0.88-0.94]	0.79	0.73	0.86	0.79	0.82
	Validation	0.82[0.74-0.89]	0.83	0.76	0.88	0.83	0.83
Model 2: the lesion including pure solid nodule							
	Training	0.78[0.74-0.82]	0.73	0.77	0.6	0.73	0.65
	Validation	0.72[0.63-0.82]	0.76	0.49	0.85	0.75	0.65

Model 2 showed good preoperative prediction performance for ADC with MPP. In the training set, AUC was 0.78 (95%CI 0.74–0.82), accuracy was 0.73, sensitivity was 0.77, specificity was 0.6, PPV was 0.73, and NPV was 0.65. In the training set, AUC was 0.76 (95% CI 0.63–0.82), accuracy was 0.76, sensitivity was 0.49, specificity was 0.85, PPV was 0.75, and NPV was 0.65.

**Figure 3:**
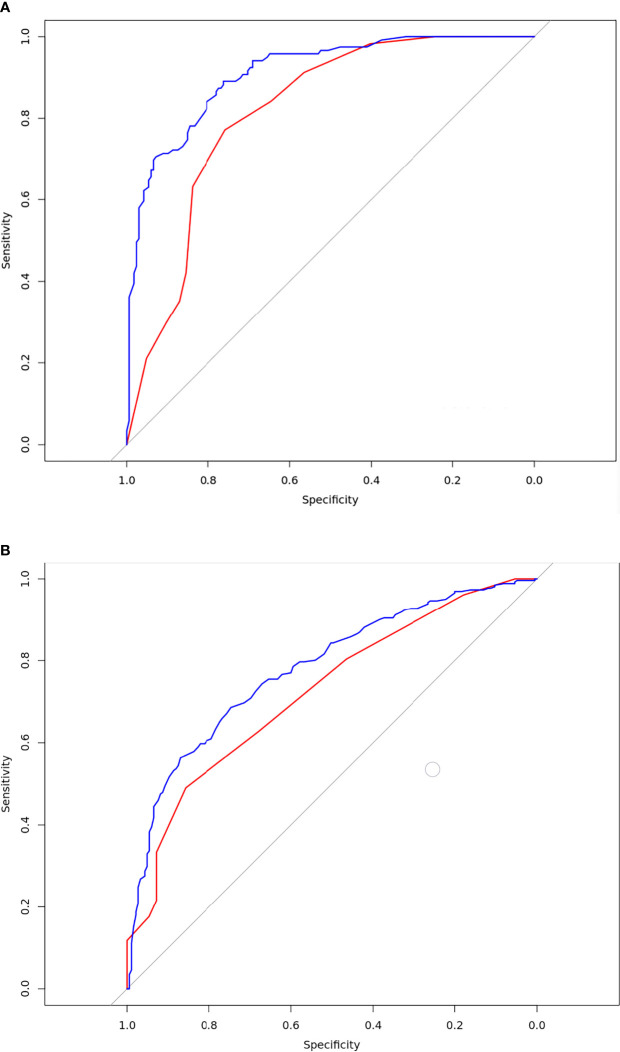
Results of the receiver-operating characteristic curve analysis for the two models. **(A)** The ROC curves for the model 1 in the training and validation database. The blue line was training set. the AUC value was 0.91[95% confidence intervals (CI):0.88-0.94]; the red line was validation set. The AUC value was 0.82[CI:0.74-0.89];Delong test p=0.0296; **(B)** The ROC curves for the model 2 in the training and validation database; The blue line was training set. The AUC value was 0.78[CI:0.74-0.82]; The red line was validation set. The AUC value was 0.72[CI:0.63-0.82];Delong test p=0.2865.

The DeLong test showed that there were statistically significant differences between the training and validation sets in model 1 (p = 0.0296) (**Figure 3A**). There was no statistically significant difference between the training and validation sets in model 2 (p = 0.2865) (**Figure 3B**).

## Discussion

In this multicenter study, stratification was based on nodule types to construct two radiomics models for preoperative prediction the MPP in peripheral lung ADC with size ≤ 2 cm. The AUC of model 1 (which included solid nodules and GGO) and model 2 (solid nodules only) were 0.91 and 0.78, respectively. Both models had good prediction ability but the prediction performance of model 2 was lower compared to that of model 1, suggesting that GGO affects prediction model performance. In the external validation set, the AUCs for the two models were 0.82 and 0.72, respectively. The DeLong test suggested a difference in AUC between the training and validation sets for model 1, showing poor generalizability. However, no significant difference was noted in AUC between the training and validation sets in model 2, showing good generalizability. Therefore, radiomics can be used as a convenient and non-invasive biomarker for preoperative prediction MPP in peripheral lung ADC with size ≤ 2 cm and to guide the diagnosis and treatment.

Previous studies proved that tumor size is an independent factor for postoperative prognosis in non-small cell lung cancer. Additionally, lung ADC with size 2.1–3 cm has a significantly higher local recurrence rate than ADC < 2 cm (21). Tumor size also affects the choice of surgical procedure for T1-stage peripheral lung ADC. Su (22) found that in ADC patients with tumor size ≤ 2 cm and MPP < 0–5%, limited resection is suitable (3, 8), whereas lobectomy may be required for patients with MPP > 5% (9). Therefore, prediction MPP in lung ADC with size ≤ 2 cm can guide surgical resection strategy.

Many previous studies have showed that CT image-based radiomics analysis can be used to predict ADC with MPP with a prediction performance of 0.7–0.98 (11–18). However, there are inconsistencies and significant differences among the results of previous studies. MPP and solid pattern appear mostly solid on CT, while solid, mGGO, and pGGO types are present in other ADC subtypes. Radiomics identification of GGO and solid lesions is simple. We speculate that the ratio of GGO and solid nodules in ADC without MPP dataset may affected the performance of the prediction model. Many previous studies using radiomics analysis combined GGO and solid nodules in the non-MPP dataset, and the proportion of GGOs was unknown. Chen (11) achieved a prediction performance of 0.86, but GGO and solid nodules accounted for 78% and 22%, respectively, in the ADC without MPP group. Park (12) obtained a prediction performance of 0.98 but the subtypes in non-MPP dataset only consisted of lepidic subtype. The prediction performance decreased to 0.84 when the dataset consisted of papillary and acinar subtype. The dataset by Wang (13) consisted of entire pGGO type for predictor MPP in ADC and the performance was below 0.80. In the current study, the AUC for the prediction performance of model 1 (solid nodules and GGO) was 0.91, but AUC decreased to 0.78 in model 2 after excluding GGO type (69%). In the final external validation, the AUC of model 1 was significantly decreased, may be due to the different ratio of GGO in the training and validation sets. Importantly, the proportion of GGO was lower in the validation set than the training set. These results suggest that the GGO type in the non-MPP dataset contributed to bias in the study results and may explain the differences in results from previous studies.

Notably, there are significant differences in the characteristics of the two prediction models. First, in model 1, the most important characteristic was mean, which was not included in model 2. Mean is a first-order characteristic related to the CT density of the lesion. The CT value of solid nodules is significantly higher than of GGO. Second, first-order characteristic parameters accounted for around half of the parameters in model 1. This may be because GGO is rare in ADC with MPP, resulting in higher contribution to first-order parameters in the histogram. In contrast, there were fewer first-order characteristics in model 2, which means that the role of density characteristics in non-solid nodule prediction is not significant. The prediction of model 1 relied more on texture features and other high-order parameters; therefore, the prediction performance of model 2 was lower than that of model 1. This emphasizes that nodule type is an important factor affecting the prediction MPP in early stage ADC, as well as the necessity of stratifying nodule type when constructing models to predict ADC with MPP. However, the proportion of solid nodules was significantly higher than GGOs (pGGO and mGGO) in ADC with MPP of size ≤ 2 cm (19). Therefore, preoperative prediction of ADC with MPP for solid nodules with 2 cm or less has great clinical value for guiding surgical treatment.

This was a multicenter study and external validation was performed for both models. The results were significantly different between the models, and the generalizability of model 2 was validated in external validation. Although the AUC of model 1 was 0.91 in the training set, which was significantly lower in external validation. There were statistically significant differences in AUC between the training set and external validation set, thereby raising concern over its generalizability for model 1. These results highlight the importance of external validation. In previous prediction studies of T1 stage ADC with MPP, only He et al. (18) performed external validation, while most others were single-center studies that lacked external validation. There are limitations to single-center studies because of over-fitting in the prediction model. Over-fitting was observed in model 1 after external validation, resulting in poor reliability of study results and limited clinical applicability. Multicenter data must be used for constructing and testing radiomics models for better clinical application.

There were several limitations to this study. First, retrospective studies have inherent weaknesses and potential bias. In future prospective works, we will strengthen the research of ratio of GGO and solid type. Second, the study included a small number of patients with MPP in ADC. Although this study included three hospitals, our sample size was small. This may be because we included patients with peripheral lung ADC with size ≤ 2 cm, and the incidence of MPP in ADC increases with tumor size. A larger sample size is required in future studies. Third, the incidence of MPP in ADC in our study is different from previous studies. As pathologists have different expertise, potential subjective deviation may be present when semi-quantitative analysis is used to record the ratio of each pathological subtype, which may affect the study results.

## Conclusion

In summary, the constructed two models based on nodule type stratification has potential to predict MPP in lung ADC of size ≤ 2 cm. We found that GGO nodule type in the without MPP dataset will affect the performance of the prediction model. Thus, the pure solid nodules (model 2) prediction had moderate stable generalizability. This model may contribute to an auxiliary method for preoperative prediction of MPP peripheral lung ADC of size ≤ 2 cm with proper treatment planning.

## Data Availability Statement

The raw data supporting the conclusions of this article will be made available by the authors, without undue reservation.

## Ethics Statement

The studies involving human participants were reviewed and approved by the First Affiliated Hospital of Zhejiang University School of Medicine; the First Affiliated Hospital of Wannan Medical College; Zhejiang Cancer Hospital. The ethics committee waived the requirement of written informed consent for participation.

## Author Contributions

ML and ZF: Study design, manuscript writing and data analysis. ML, YR, and FS: labeled the image. LZ and MW: CT raw data collection. All authors contributed to the article and approved the submitted version.

## Conflict of Interest

The authors declare that the research was conducted in the absence of any commercial or financial relationships that could be construed as a potential conflict of interest.

## Publisher’s Note

All claims expressed in this article are solely those of the authors and do not necessarily represent those of their affiliated organizations, or those of the publisher, the editors and the reviewers. Any product that may be evaluated in this article, or claim that may be made by its manufacturer, is not guaranteed or endorsed by the publisher.
